# Mitigation of influenza-mediated inflammation by immunomodulatory matrix-bound nanovesicles

**DOI:** 10.1126/sciadv.adf9016

**Published:** 2023-05-19

**Authors:** Raphael J. Crum, Brydie R. Huckestien, Gaelen Dwyer, Lisa Mathews, David G. Nascari, George S. Hussey, Heth R. Turnquist, John F. Alcorn, Stephen F. Badylak

**Affiliations:** ^1^ McGowan Institute for Regenerative Medicine, University of Pittsburgh, Pittsburgh, PA, USA.; ^2^ Division of Pulmonary Medicine, Department of Pediatrics, UPMC Children’s Hospital of Pittsburgh, Pittsburgh, PA, USA.; ^3^ Department of Immunology, University of Pittsburgh, Pittsburgh, PA, USA.; ^4^ Thomas E. Starzl Transplantation Institute, University of Pittsburgh, Pittsburgh, PA, USA.; ^5^ Department of Surgery, School of Medicine, University of Pittsburgh, Pittsburgh, PA, USA.; ^6^ Department of Pathology, School of Medicine, University of Pittsburgh, Pittsburgh, PA, USA.; ^7^ Department of Bioengineering, University of Pittsburgh, Pittsburgh, PA, USA.

## Abstract

Cytokine storm describes a life-threatening, systemic inflammatory syndrome characterized by elevated levels of proinflammatory cytokines and immune cell hyperactivation associated with multi-organ dysfunction. Matrix-bound nanovesicles (MBV) are a subclass of extracellular vesicle shown to down-regulate proinflammatory immune responses. The objective of this study was to assess the efficacy of MBV in mediating influenza-induced acute respiratory distress syndrome and cytokine storm in a murine model. Intravenous administration of MBV decreased influenza-mediated total lung inflammatory cell density, proinflammatory macrophage frequencies, and proinflammatory cytokines at 7 and 21 days following viral inoculation. MBV decreased long-lasting alveolitis and the proportion of lung undergoing inflammatory tissue repair at day 21. MBV increased the proportion of activated anti-viral CD4^+^ and CD8^+^ T cells at day 7 and memory-like CD62L^+^ CD44^+^, CD4^+^, and CD8^+^ T cells at day 21. These results show immunomodulatory properties of MBV that may benefit the treatment of viral-mediated pulmonary inflammation with applicability to other viral diseases such as SARS-CoV-2.

## Teaser

Matrix-bound nanovesicles mitigate influenza-mediated inflammation and may be therapeutic in treating viral-driven cytokine storm.

## INTRODUCTION

Cytokine storm is a generic term used that describes a life-threatening, systemic inflammatory syndrome characterized by elevated levels of proinflammatory cytokines and immune cell hyperactivation associated with multi-organ dysfunction and failure. The etiology of cytokine storm is multifactorial and not fully understood but includes adverse responses to cancer, autoimmune disease, respiratory pathogens such as influenza, and various therapeutic agents (*1*, *2*). Interest in the topic of cytokine storm has increased because of the severe acute respiratory syndrome coronavirus 2 (SARS-CoV-2) and the associated pandemic. The SARS-CoV-2 pandemic, similar to the H1N1 pandemics of 1918 and 2009, has been characterized by a variety of signs and symptoms ranging from mild fatigue to life-threatening pneumonia, respiratory distress, cytokine storm, multi-organ failure, and viral-induced acute respiratory distress syndrome (ARDS). The SARS-CoV-2- and H1N1-associated cytokine storm and ARDS are characterized by increased serum proinflammatory cytokines such as interleukin-1β (IL-1β), IL-6, tumor necrosis factor–α (TNF-α), and interferon-γ (IFN-γ) (*3*–*12*). In addition to these elevated cytokine levels, the relative frequencies of activated CD4^+^ and CD8^+^ T cells are substantially increased in patients with SARS-CoV-2 (*13*, *14*). In more severe cases of SARS-CoV-2, T cell lymphopenia is present (*15*, *16*). The combination of hyperinflammatory changes in systemic cytokines and activated immune cells with associated tissue damage is predictive of worse outcomes from viral-induced ARDS (*3*–*5*, *7*–*9*, *17*).

Homeostasis of health in all tissues requires a finely tuned balance between the proinflammatory and anti-inflammatory or regulatory components of the immune system. Dysregulation of this immune balance, as seen in states of hyperinflammation and cytokine storm, results in pathology. The regulation of tissue immune homeostasis involves complex immune-stromal cross-talk that is influenced by the extracellular matrix (ECM). The recent identification of a distinct population of immunomodulatory nanovesicles embedded within the ECM provides insight into one potential mechanism by which tissue immune homeostasis is maintained (*18*–*22*). These nanovesicles, identified as matrix-bound nanovesicles (MBVs), are a distinct subclass of extracellular vesicle (EV) that are different from fluid-phase exosomes and are enriched in anti-inflammatory and immunoregulatory-associated microRNA, proteins, and lipids (*18*–*20*). Previous studies of administered MBV show their ability to down-regulate proinflammatory immune responses and improve outcomes in animal models of acute ocular injury, rheumatoid arthritis, and chronic heart transplant rejection (*23*–*25*). In addition, MBVs have been shown to have minimal systemic and immune toxicity in vivo and biodistribution to immune tissues such as the spleen through various routes of administration (*26*). This immunomodulatory capacity is mediated, at least in part, via mitigation of proinflammatory macrophages and promotion of an anti-inflammatory macrophage phenotype without apparent loss of immune competence.

Given the lack of any reliably effective therapies, the objective of the present study was to assess the potential therapeutic efficacy of administered MBV against H1N1-induced ARDS and the associated cytokine storm in a murine model. Influenza H1N1 induces similar lung injury, cytokine storm, and viral-induced ARDS to that observed in SARS-CoV-2 patients, albeit with differing degrees of specific immune pathway activation. Furthermore, H1N1 follows a similar pathogenic time course, including rapid ARDS-like lung injury and persistent alveolitis for weeks or months. MBV were delivered systemically to mice following intranasal inoculation with H1N1 to assess both the therapeutic efficacy of MBV treatment and the immunomodulatory properties of MBV in an established model of viral-mediated pulmonary inflammation and systemic cytokine storm. The findings of the present study show that the immunomodulatory properties of MBV may have a therapeutic benefit in the treatment of influenza-mediated pulmonary inflammation with applicability to other viral-mediated inflammatory diseases such as SARS-CoV-2.

## RESULTS

### Systemic administration of MBV mitigates acute viral-mediated pulmonary inflammation through inhibition of mononuclear cell infiltration and down-regulation of proinflammatory chemokines and cytokines

**Fig. 1.**
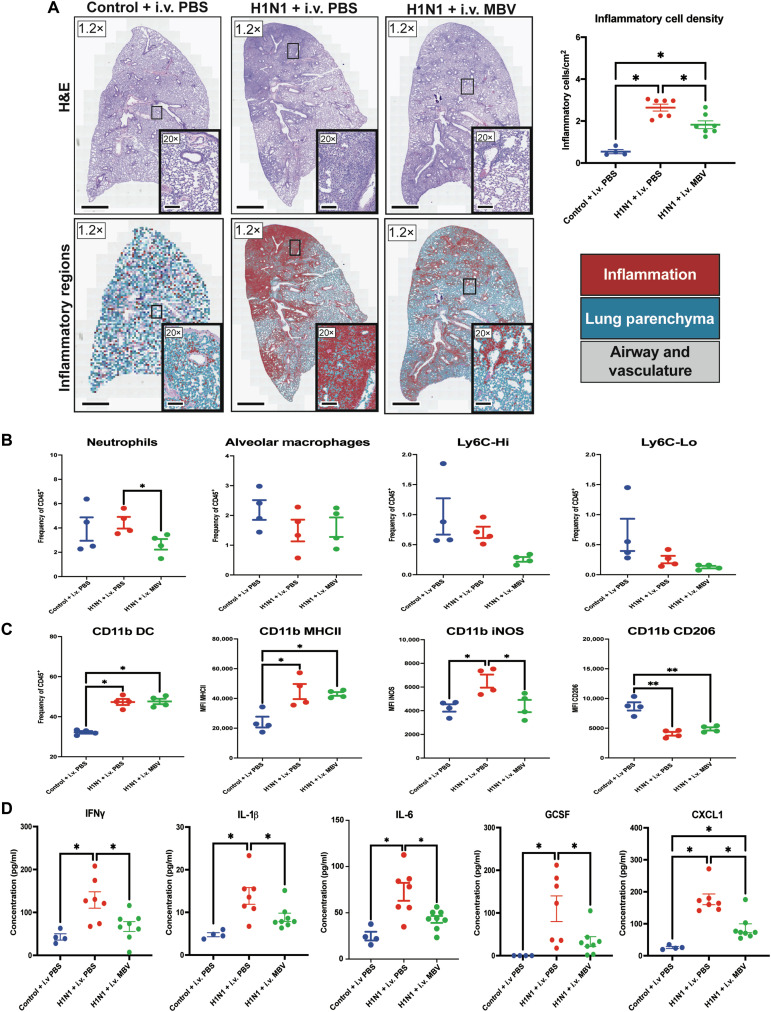
Systemic administration of MBV inhibits acute (day 7) viral-mediated pulmonary inflammation through decreased tissue cellular infiltration and decreased expression of proinflammatory chemokines and cytokines. (**A**) Representative H&E slide scans (1.2×) and QuPath rendered images of lungs with higher magnification images (20×) for each experimental group at day 7 following intranasal inoculation of A PR/8/34 H1N1 strain. QuPath-derived cell classification highlights healthy lung parenchyma (blue), inflammatory lung parenchyma (red), and airways and vasculature in gray. Scale bars for 1.2× images, 1000 μm. Scale bars for 20× images, 200 μm (values = means ± SEM; *n* = 4 for Control + PBS, *n* = 7 for H1N1 + intravenous (i.v.) PBS, and *n* = 7 for H1N1 + MBV; significant differences *P < 0.05). (**B**) Frequency of lung tissue neutrophils, alveolar macrophages, Ly6C-Hi, and Ly6C-Lo monocytes relative to total CD45^+^ cells as detected by flow cytometry (values = means ± SEM; *n* = 4; significant differences *P < 0.05). (**C**) Frequency of lung CD11b + dendritic cells (DC) and MFI of MHCII, iNOS, and CD206 expression in the lung CD11b + DC population (values = means ± SEM; *n* = 4; significant differences *P < 0.05). (**D**) Concentration of proinflammatory IFN-γ, IL-1β, IL-6, GCSF, and CXCL1 in the lung tissue homogenates as determined by cytokine multiplex (values = means ± SEM; *n* = 4 for Control + PBS, *n* = 7 for H1N1 + intravenous PBS, and *n* = 8 for H1N1 + MBV; significant differences *P < 0.05).

Intravenous administration of MBV at days 2 and 6 following viral inoculation at day 0 showed marked reduction in acute H1N1-mediated pulmonary pathology at day 7. At day 7, vehicle-treated animals [H1N1 + intravenous PBS (phosphate-buffered saline)] developed acute viral-mediated pulmonary inflammation characterized by a dense interstitial cellular infiltrate consisting of mononuclear cells and lymphocytes, thickening of the alveolar septa and associated alveolar collapse, and accumulation of proteinaceous exudate. Using quantitative, artificial intelligence (AI)–assisted pathology analysis, the number of inflammatory cells per square millimeter was increased in the H1N1 + intravenous PBS group compared to the Control + intravenous PBS group [2.64 ± 0.17 cells/mm^2^ versus 0.54 ± 0.09 cells/mm^2^ (*P* < 0.05); Fig. 1A]. Animals treated with intravenous MBV (H1N1 + intravenous MBV) showed a reduction in interstitial inflammation and cell density compared to the H1N1 + intravenous PBS group [1.83 ± 0.19 cells/mm^2^ versus 2.64 ± 0.17 cells/mm^2^ (*P* < 0.05); Fig. 1A]. Disease severity was assessed by viral burden, bronchoalveolar lavage (BAL) protein secretion, IFN-β- and IFN-λ-related geneexpression, epithelial-related gene expression, and weight loss across the study. Despite the observed decreases in interstitial inflammation and cellularity, no differences were observed between the H1N1 + intravenous PBS and H1N1 + intravenous MBV group in all four metrics (*P* > 0.05; fig. S2).

Flow cytometry analysis (fig. S1) of lung tissue showed a significant reduction in the total frequency of neutrophils (defined as CD45^+^ CD11bHi Ly6G^+^ cells) in the lung in H1N1 + intravenous MBV animals compared to H1N1 + intravenous PBS during acute inflammation (*P* < 0.05; Fig. 1B). Across all groups, no differences were observed in the frequency of alveolar macrophages (CD45^+^ CD11b-Int SiglecF^+^ cells), LyC6-Hi monocytes, or Ly6-Clo monocytes (*P* > 0.05; Fig. 1B). While both H1N1 + intravenous PBS and H1N1 + intravenous MBV significantly increased the frequency of CD11b myeloid dendritic cells and mean fluorescent intensity (MFI) of CD11b major histocompatibility complex II (MHCII) in the lung (*P* < 0.05), there were no differences between those two groups in both metrics (*P* > 0.05; Fig. 1C). Further interrogation of the CD11b subset showed a significant reduction in the MFI of inducible nitric oxide synthase (iNOS) expressing CD11b myeloid dendritic cells in the lung tissue in H1N1 + intravenous MBV treatment compared to H1N1 + intravenous PBS (*P* < 0.05; Fig. 1C). The MFI of CD206 expressing CD11b dendritic cells was significantly decreased in both the H1N1 + intravenous PBS and H1N1 + intravenous MBV groups relative to the Control + intravenous PBS group (*P* < 0.05; Fig. 1C). These data suggest that systemic MBV delivery after H1N1 infection can reduce lung pathology, which is associated with decreased lung infiltration of proinflammatory neutrophils and iNOS-expressing myeloid antigen-presenting cells.

In addition to differences observed in the cellular infiltration of lungs within the first week, profound differences were noted between groups in the production of proinflammatory cytokines and chemokines in the lung tissue. Specifically, IL-6, IL-1β, TNF-α, IFN-γ, granulocyte colony stimulating factor (GCSF), and CXCL1 were down-regulated in the lung tissue of animals treated with intravenous MBV compared to the H1N1 + intravenous PBS animals (*P* < 0.05; Fig. 1D). No differences were observed between the intravenous MBV group and the Control + intravenous PBS group regarding these proinflammatory cytokines and chemokines (*P* > 0.05; Fig. 1D), and similarly, no differences were found between the H1N1 + intravenous PBS and the H1N1 + intravenous MBV group in the remaining cytokines and chemokines included in the cytokine multiplex assay at day 7 (table S1). Thus, MBV were effective at reducing proinflammatory chemokines and cytokines in the lung during H1N1 infection.

### MBV drives an increase in the ratio of activated CD8^+^:CD4^+^ T cells in both the lung and spleen favoring an antiviral response in the acute phase of inflammation

**Fig. 2.**
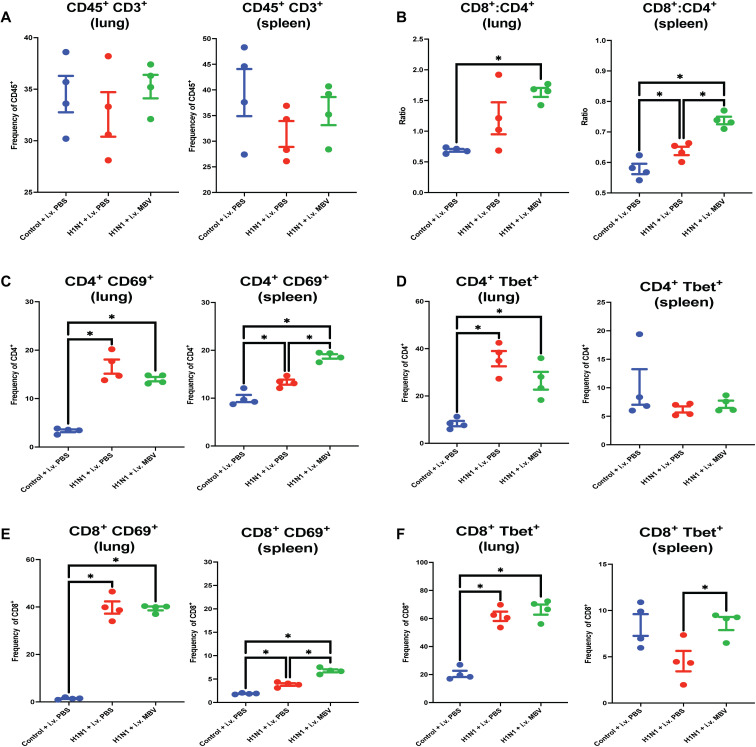
Intravenous administration of MBV increases in the local and systemic ratio of activated CD8^+^:CD4^+^ T cells in the acute phase (day 7) of H1N1-mediated airway inflammation. (**A**) Frequency of CD45^+^ CD3^+^ T cells in both the lung and spleen relative to total CD45^+^ cells. (**B**) Ratio of CD8^+^:CD3^+^ T cell frequency to CD4^+^ CD3^+^ T cell frequency (of total CD45^+^ cells) in both the lung and spleen. (**C**) Frequency of CD4^+^ CD69^+^ T cells in both the lung and spleen relative to total CD4^+^ cells. (**D**) Frequency of CD4^+^ Tbet^+^ T_H_1 cells in both the lung and spleen relative to total CD4^+^ cells. (**E**) Frequency of CD8^+^ CD69^+^ T cells in both the lung and spleen relative to total CD8^+^ cells. (**F**) Frequency of CD8^+^ Tbet^+^ T cells in both the lung and spleen relative to total CD8^+^ cells. All values = means ± SEM; *n* = 4; significant differences *P < 0.05. i.v., intravenous.

In the acute phase of influenza-mediated inflammation, systemic administration of MBV had both local and systemic effects on T cell populations. In both the lung and spleen, there were no differences across all three groups regarding the total frequency of CD45^+^ CD3^+^ T cells (*P* > 0.05; Fig. 2A), but MBV administration resulted in a significant increase in the ratio of CD8^+^:CD4^+^ T cells compared to the Control + intravenous PBS group (*P* > 0.05; Fig. 2B). In the spleen, MBV treatment increased the ratio of CD8^+^:CD4^+^ T cells compared to the H1N1 + intravenous PBS group (*P* > 0.05; Fig. 2B).

In both the lung and spleen, CD4^+^ T cell activation (CD69^+^) was increased by both the H1N1 + MBV and H1N1 + intravenous PBS groups compared to the Control + intravenous PBS animals (*P* < 0.05; Fig. 2C). In the spleen, MBV administration increased the proportion of CD4^+^ CD69^+^ T cells relative to H1N1 + intravenous PBS group (*P* < 0.05; Fig. 2C). In the lung, CD4^+^ Tbet^+^ T helper 1 (T_H_1) T cells were increased in both the H1N1 + intravenous PBS and H1N1 + intravenous MBV groups relative to the Control + intravenous PBS group (*P* < 0.05; Fig. 2D). No differences in CD4^+^ Tbet^+^ T_H_1 T cells were shown among the three groups (*P* < 0.05; Fig. 2D). Both the H1N1 + intravenous PBS and H1N1 + intravenous MBV groups showed increased frequency of CD8^+^ CD69^+^ T cells in the lung (*P* < 0.05; Fig. 2E). Likewise, the H1N1 + intravenous PBS and H1N1 + intravenous MBV groups significantly increased the frequency of CD8^+^ CD69^+^ T cells in the spleen (*P* < 0.05; Fig. 2E). Furthermore, the H1N1 + intravenous MBV significantly increased the frequency of CD8^+^ CD69^+^ T cells in the spleen relative to the H1N1 + intravenous PBS group (*P* < 0.05; Fig. 2E). MBV administration significantly increased the frequency of activated CD69^+^ CD4^+^ and CD69^+^ CD8^+^ T cells compared to intravenous PBS–treated animals. Similar to what was observed in the frequencies of CD4^+^ Tbet^+^ T cells, both H1N1 + intravenous PBS and H1N1 + intravenous MBV groups increased the frequency of CD8^+^ Tbet^+^ T cells compared to the Control + intravenous PBS group (*P* < 0.05; Fig. 2F). In the spleen, however, the frequency of CD8^+^ Tbet^+^ T cells was increased in the H1N1 + intravenous MBV groups compared to the H1N1 + intravenous PBS group (*P* < 0.05; Fig. 2F). These data showing increased splenic T cell activation are consistent with an MBV-augmented systemic immune response to H1N1.

### Systemic administration of MBV induces sustained down-regulation proinflammatory cytokines and chemokines with a decrease in total lung cell infiltration during chronic lung injury and repair at day 21

**Fig. 3.**
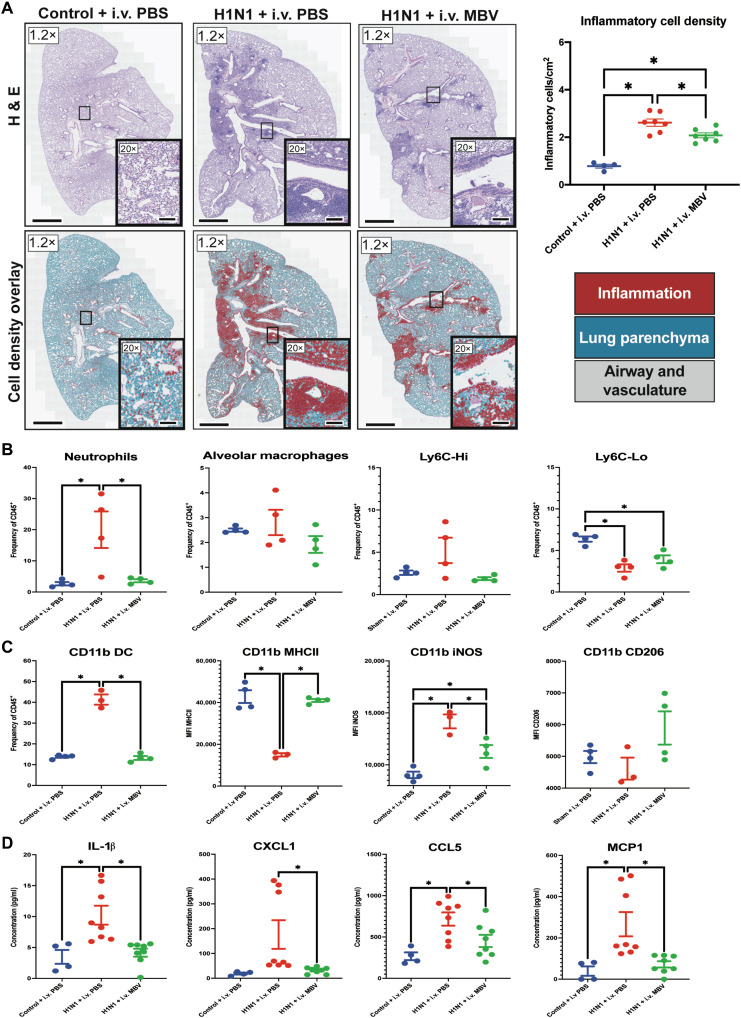
Systemic administration of MBV inhibits chronic (day 21) viral-mediated pulmonary inflammation through decreased tissue cellular infiltration and decreased expression of proinflammatory chemokines and cytokines. (**A**) Representative H&E slide scans (1.2×) and QuPath rendered images of lungs with higher magnification images (20×) for each experimental group at day 7 following intranasal inoculation of A PR/8/34 H1N1 strain. QuPath-derived cell classification highlights healthy lung parenchyma (blue), inflammatory lung parenchyma (red), and airways and vasculature in gray. Scale bars for 1.2× images, 1000 μm. Scale bars for 20× images, 200 μm (values = means ± SEM; *n* = 4 for Control + PBS, *n* = 7 for H1N1 + intravenous (i.v.) PBS, and *n* = 7 for H1N1 + MBV; significant differences *P < 0.05). (**B**) Frequency of lung tissue neutrophils, alveolar macrophages, Ly6C-Hi, and Ly6C-Lo monocytes relative to total CD45^+^ cells as detected by flow cytometry (values = means ± SEM; *n* = 4; significant differences *P < 0.05). (**C**) Frequency of lung CD11b + dendritic cells (DC) and MFI of MHCII, iNOS, and CD206 expression in the lung CD11b + DC population (values = mean ± SEM; *n* = 4; significant differences *P < 0.05). (**D**) Concentration of proinflammatory IL-1β, CXCL1, CCL5, and MCP1 in the lung tissue homogenates as determined by multiplexed enzyme-linked immunosorbent assay (values = means ± SEM; *n* = 4 for Control + PBS, *n* = 7 for H1N1 + intravenous PBS, and *n* = 8 for H1N1 + MBV; significant differences *P < 0.05).

Systemic administration of MBV, delivered twice during the first week of H1N1 infection, resulted in a sustained immunomodulatory effect through 21 days following inoculation. The H1N1 + intravenous PBS animals at day 21 showed a maturation of the early diffuse interstitial and alveolar predominant inflammation at day 7 to a more focal disseminated inflammatory response localizing to the peribronchiolar region (Fig. 3A, H1N1 + intravenous PBS). The number of inflammatory cells per square millimeter was again increased in the H1N1 + intravenous PBS group compared to the Control + intravenous PBS group [2.62 ± 0.15 cells/mm^2^ versus 0.78 ± 0.08 cells/mm^2^ (*P* < 0.05); Fig. 3A]. Animals treated with intravenous MBV (H1N1 + intravenous MBV), however, showed a reduction in interstitial inflammation and cell density compared to the H1N1 + intravenous PBS group [2.08 ± 0.10 cells/mm^2^ versus 2.62 ± 0.15 cells/mm^2^ (*P* < 0.05); Fig. 3A]. There were no significant differences observed in viral burden, BAL protein secretion, IFN-β- and IFN-λ-related gene expression, epithelial-related gene expression, and weight loss at day 21 after infection between the H1N1 + intravenous PBS and H1N1 + intravenous MBV groups (*P* > 0.05; fig. S3).

Similar to what was observed at day 7, MBV administration significantly reduced the frequency of neutrophils (CD45^+^ CD11bHi Ly6G^+^) in the lung compared to the H1N1 + intravenous PBS animals at day 21 (*P* < 0.05; Fig. 3C). There were no differences among all three groups in the frequency of alveolar macrophages (CD11b-Int SiglecF^+^) and Ly6C-Hi monocytes (CD11lb-Hi CD64- MHCII-) in the lung (*P* < 0.05; Fig. 3B). The frequency of Ly6C-Lo monocytes was decreased in both the H1N1 + intravenous PBS and H1N1 + intravenous MBV groups compared to the Control + intravenous PBS group (*P* < 0.05; Fig. 3B). There was no difference in the frequency of Ly6C-Lo monocytes observed between the H1N1 + intravenous PBS and H1N1 + intravenous MBV groups (*P* > 0.05). In addition, MBV treatment significantly reduced the frequency of CD11b dendritic cells (CD11b^+^ CD11c^+^) in the lung tissue (*P* < 0.05; Fig. 3C). In the CD11b dendritic cells, the MFI of MHCII was increased in the MBV-treated animals, and the MFI of iNOS was decreased compared to the H1N1 + intravenous PBS group (*P* < 0.05; Fig. 3C). Across all groups, there were no differences in the MFI of CD206 on CD11b dendritic cells (*P* < 0.05; Fig. 3C). In addition to differences observed in the cellular infiltration of lungs at day 21, significant differences were observed in the production of proinflammatory cytokines and chemokines in the lung tissue. Specifically, IL-1β, CXCL1, CCL5, and MCP1 were down-regulated in the lung tissue of those animals treated with intravenous MBV compared to the vehicle-treated animals (*P* < 0.05; Fig. 3D). No differences were observed between the H1N1 + intravenous MBV group and the Control + intravenous PBS group regarding these proinflammatory cytokines and chemokines (*P* < 0.05; Fig. 3D). There were no differences observed between the H1N1 + intravenous PBS and the H1N1 + intravenous MBV groups in the remaining cytokines and chemokines included in the multiplex assay at day 21 (table S2). Thus, early MBV treatment is effective at reducing neutrophils and proinflammatory chemokines and cytokines to baseline levels in the lung by 21 days after infection.

### MBV up-regulates systemic populations of memory-like CD4^+^ CD62L^+^ CD44^+^ and CD8^+^ CD62L^+^ CD44^+^ T cells

**Fig. 4.**
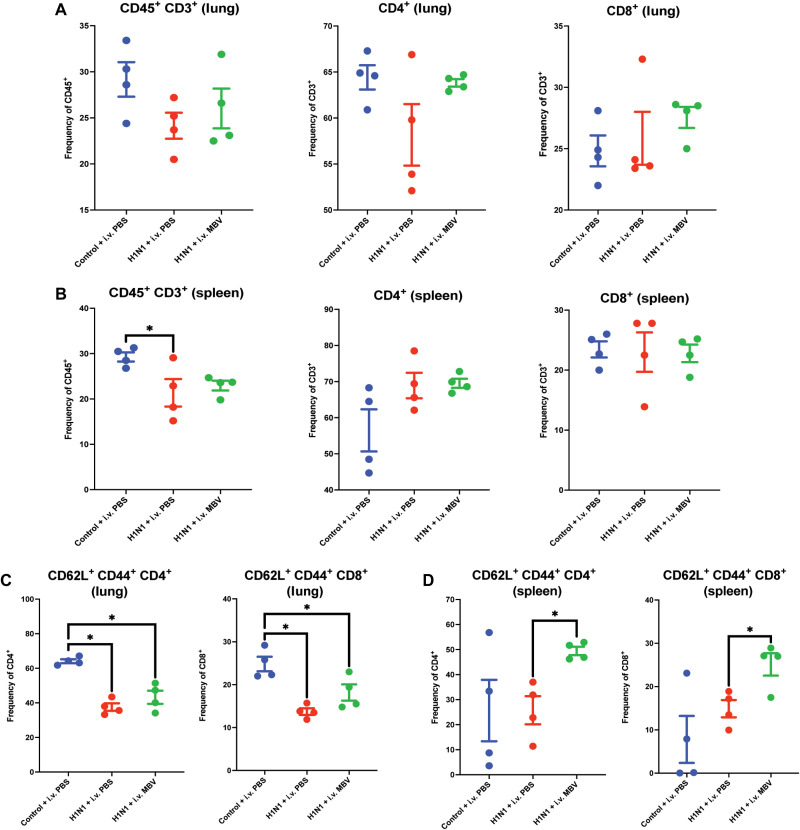
MBV up-regulates splenic populations of memory-like CD4^+^/CD62L^+^/CD44^+^ and CD8^+^/CD62L/CD44^+^ T cells. (**A**) Frequency of CD45^+^ CD3^+^ T cells, CD4^+^ T cells, and CD8^+^ T cells in the lung relative to total CD45^+^ cells. (**B**) Frequency of CD45^+^ CD3^+^ T cells, CD4^+^ T cells, and CD8^+^ T cells in the spleen relative to total CD45^+^ cells. (**C**) Frequency of CD4^+^ and CD8^+^ CD44^+^ CD62L^+^ cells in the lung. (**D**) Frequency of CD4^+^ and CD8^+^ CD44^+^ CD62L^+^ cells in the spleen. All values = means ± SEM; *n* = 4; significant differences *P < 0.05. i.v., intravenous.

MBV administration showed both local and systemic effects on both CD4^+^ and CD8^+^ T cell populations similar to that observed at day 7. There were no differences in the frequency of CD3^+^, CD4^+^ or CD8^+^ T cells between the lung and spleen (*P* > 0.05; Fig. 4, A and B). However, MBV appeared to exhibit a potent effect on T cell memory at this time point as determined by CD62L and CD44 expression on CD4^+^ and CD8^+^ T cells. The frequencies of CD4^+^ CD44^+^ CD62L^+^ and CD8^+^ CD44^+^ CD62L^+^ presumed that central memory T cells were not altered in the lung by MBV treatment at day 21 after infection (*P* > 0.05; Fig. 4C). However, phenotypical central memory CD4^+^ CD44^+^ CD62L^+^ and CD8^+^ CD44^+^ CD62L^+^ T cells frequencies were significantly increased in the spleen by intravenous MBV compared to the H1N1 + PBS group (*P* > 0.05; Fig. 4D). In addition, HAI antibody titers were assessed to evaluate B cell–mediated antibody responses, and while MBV reduced the titer dilution factor, the difference between H1N1 + intravenous PBS and H1N1 + intravenous MBV was not significant (*P* = 0.09; fig. S4). Thus, MBVs were suggested to support immunity after viral infection while limiting tissue pathology and myeloid inflammation.

### Acute administration of MBV decreases long-lasting alveolitis and the proportion of total lung tissue showing inflammatory-mediated tissue repair

**Fig. 5.**
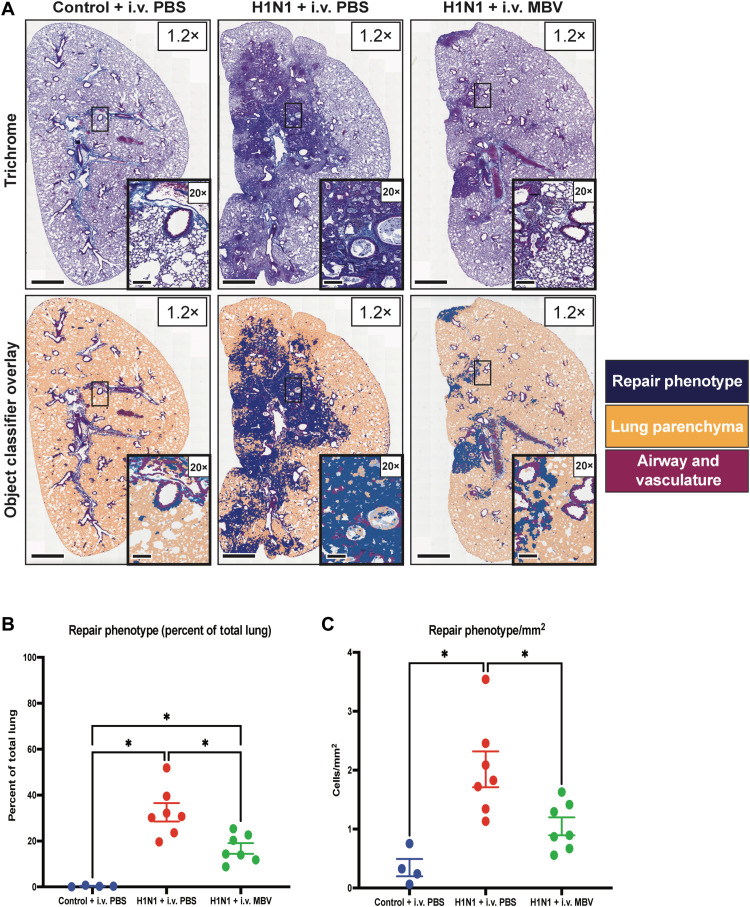
MBV administration during the acute phase of H1N1-mediated pulmonary inflammation decreases long-lasting alveolitis and the proportion of total lung tissue undergoing inflammatory-mediated tissue repair. (**A**) Representative whole-lung images of Masson’s trichrome-stained lung sections (1.2×) and QuPath-classified images of lungs with higher magnification images (20×) for each experimental group at day 21 following intranasal inoculation of A PR/8/34 H1N1 strain. A QuPath AI–assisted object classifier pipeline was trained to identify “healthy” lung parenchyma (orange) and “disease-associated” repair phenotype (blue) and to exclude airway and vascular structures (maroon) from any quantification. Scale bars for 1.2× images, 1000 μm. Scale bars for 20× images, 200 μm. (**B**) Using QuPath and the previously described object classifier, the percent of the total lung tissue surface area involved in the repair phenotype was quantified for all groups. (**C**) Combining the QuPath classifier and cell density measurements, the number of cells classified as repair phenotype were quantified per square millimeter (cells/mm^2^) (values = means ± SEM; *n* = 4 for Control + PBS, *n* = 7 for H1N1 + intravenous (i.v.) PBS, and *n* = 8 for H1N1 + MBV; significant differences *P < 0.05). In total, MBV administration early during H1N1 infection provided protection against persistent inflammatory-mediated tissue repair.

Chronic, unresolving bronchiolitis and alveolitis can result in progression toward a long-term injury state characterized by degeneration and desquamation of lung epithelium followed by excess epithelial proliferation and mucous cell metaplasia. While these processes are indicative of tissue repair to viral insult, sustained and increased epithelial injury has long-term consequences in the lung (*27*). The development of sustained lung repair phenotype was identified by using Masson’s trichrome staining of lung sections and a QuPath, AI-assisted algorithm to classify healthy and repair phenotype tissue (Fig. 5A). In the H1N1 + intravenous PBS animals, 21 days after viral inoculation, chronic and persistent inflammation resulted in both an increase in the proportion of total lung involved in a repair phenotype (*P* < 0.05; Fig. 5B) and an increase density of repair-associated cells per square millimeter of tissue (*P* < 0.05; Fig. 5C).

The repair phenotype in the H1N1 + intravenous PBS animals was primarily peribronchiolar in distribution with additional involvement surrounding large airway structures. The repair phenotype did not extend to all margins of the lung parenchyma. MBV administration reduced the percentage of repair phenotype throughout the lung parenchyma compared to the untreated, H1N1 + intravenous PBS group (*P* < 0.05; Fig. 5B). While the repair phenotype in the MBV-treated group shared a similar focal pattern around the larger airways, there was a marked reduction in extension of this phenotype to the margins of the lung parenchyma. In addition, MBV treatment significantly reduced the density of repair phenotype tissue per square millimeter compared to the H1N1 + intravenous PBS animals (*P* < 0.05; Fig. 5C).

## DISCUSSION

Substantial clinical and preclinical investigations have been focused on the identification of potential therapeutic agents that mitigate the acute and chronic sequelae of influenza-associated airway inflammation, cytokine storm, and viral-induced ARDS. One of the major systemic sequelae of viral-mediated pulmonary disease is the increase in systemic inflammatory cytokine levels (*28*, *29*). Treatment strategies for this systemic increase in proinflammatory cytokine (the cytokine storm) have primarily focused on supportive care to maintain critical organ function in combination with both nonspecific and targeted immunosuppression (*3*, *30*). Current options include broad-spectrum immunosuppressants such as corticosteroids and targeted biologics such as cytokine decoy receptors and signaling inhibitors. While immunosuppressive drugs have been shown to be beneficial in mitigating acute symptoms, pathologic hyperinflammation in the cytokine storm syndrome is itself a state of immunodeficiency that, in combination with immunosuppressive medication, places patients at increased risk for immunosuppression-associated sequelae. Therefore, there exists a need for immunomodulatory therapies that facilitate a host response to viral infection without compromising the entire immune system.

The immunomodulatory and therapeutic efficacy of systemically administered MBV was assessed in a murine model of H1N1-mediated pulmonary inflammation. Results showed that systemic administration of MBV reduced lung inflammation, lung tissue damage, and concentration of inflammatory mediators following H1N1 exposure. Specifically, MBV decreased the frequency of lung-invading neutrophils and proinflammatory cytokines associated with immune cell recruitment and activation (IFN-γ, IL-1β, Il-6, GCSF, CXCL1, CCL5, and MCP1). While neutrophils function as part of the innate immune system to resolve viral infection, the inflammatory consequence of excess neutrophil tissue invasion and release of proinflammatory cytokines and chemokines has been shown to compromise prognosis in viral-associated pulmonary inflammation (*31*–*39*). Results of the present study show that MBV reduced the total frequency of neutrophils at days 7 and 21 compared to the untreated animals. This decreased innate immune response was corroborated by a corresponding decrease in IFN and nucleotide-binding oligomerization domain (NOD)–related mediators. In the acute phase of inflammation, IFN-γ was decreased with MBV treatment and was accompanied by a decrease in inflammatory mediators including IL-1β and CXCL1 at day 7 and IL-1β, CXCL1, and CCL5 at day 21. While IFN-mediated signaling is associated with a robust innate response to viral pathogen, there has been evidence that the inhibition of IFN-γ–signaling through IFN-γ receptor knockdown experiments attenuates H1N1-mediated pulmonary inflammation (*40*–*43*).

Consistent with down-regulation of IFN-γ–related signaling, MBV limited the expression of iNOS in CD11b-expressing myeloid dendritic cells in the lung tissue in the present study. MBV have been shown to have a similar effect on iNOS-expressing, M1-like cells in vitro and in vivo in a model of rheumatoid arthritis (*19*, *21*, *23*). While these results have been consistently observed after in vitro and in vivo MBV treatment, the specific mechanisms of action remain unknown, and the identification of such mechanisms should be the subject of future studies.

In addition to the MBV-mediated effect observed on cells of myeloid origin, MBV were shown to promote early resolution of the inflammation systemically in the spleen at day 7 by supporting the function of lymphoid cells. This support was shown by the increased CD8^+^:CD4^+^ ratio, increased CD4^+^ and CD8^+^ activation (CD69^+^ expression), and increased frequency of type 1, Tbet^+^-expressing CD8^+^ T cells. The shift in relative CD8^+^:CD4^+^ T cell ratio induced by MBV treatment was accompanied by an increase in the spleen of CD8^+^ CD69^+^ (activated CD8^+^ T cells) and Tbet^+^ CD8^+^ T cells. Considered together, this shift toward an activated and type-1 anti-viral CD8^+^ T cell response may have contributed to rapid mitigation of the early viral inflammation, an occurrence that ultimately resulted in decreased lung injury with MBV treatment seen in the trichrome histology at day 21. Previous studies have shown that a robust activated CD8^+^ Tbet^+^ response is essential for viral clearance and resolution of viral-mediated pulmonary inflammation (*44*–*47*). Furthermore, systemic administration of MBV was shown in the present study to increase the frequency of memory-like, CD44^+^ CD62L^+^ CD4^+^ and CD8^+^ T cells in the spleen of animals 21 days after viral inoculation, a finding that suggests the possibility of an effective future antiviral responses to reinfection (*48*–*50*).

The present study complements a growing interest in the application of EV-based therapies for managing viral-mediated cytokine storm and ARDS. Since the beginning of the SARS-CoV-2 pandemic between late 2019 and early 2020, there has been a rapid acceleration in the clinical translation of EV therapies targeting aberrant inflammatory networks in viral-mediated pulmonary inflammation. To date, there are 10 current and active clinical trials investigating EV-based therapies for SARS-CoV-2 (*51*). While these trials are primarily focused on safety and pharmacodynamics of EV therapies, there is growing evidence to support the immunomodulatory role of EV therapies in managing viral-mediated inflammatory lung pathology (*52*–*56*).

Although the results of the present study show early evidence for the immunomodulatory potential of MBV in mitigating aberrant cytokine storm and pulmonary inflammation following H1N1 infection, the specific molecular mechanism(s) for this immunomodulation is/are not fully understood. While MBV used in this study were isolated from porcine urinary bladder, recent studies have shown that MBV cargo and bioactivity varies depending on tissue source (*57*). It is possible that the immunomodulatory benefits observed with MBV in this study could have been improved using MBV isolated from a different tissue source such as lung tissue (*56*). The present study showed significant changes in immune cell proportions in the lung and spleen, results that were not associated with improvement in body weight or gene expression values classically associated with improved antiviral response, as the viral dose used was nonlethal. Future studies are necessary to determine whether the immunomodulatory effect of MBV confers an additional mortality benefit in an acute and lethal viral titer of H1N1. The immunophenotyping results in the present study were also limited by the absence of B cell titer data and antigen-specific memory T cell expansion. Furthermore, the treatment paradigm investigated in this study was analogous to that of influenza antiviral neuraminidase inhibitors, such as oseltamivir and zanamivir, that are indicated for treatment in the early phase of viral infection and inflammation (*58*). The therapeutic potential of MBV administered outside the initial infection and acute inflammatory window was not investigated. Therefore, future studies are required to evaluate MBV therapy for influenza in the later stages of disease as patients present clinically at varied stages and severities of their respiratory illness. In addition, future investigation into the in vivo biodistribution of MBV in the context of tissue inflammation is warranted. While previous studies have shown the biodistribution of MBV in nondiseased animal models, future studies are necessary to clarify whether MBV biodistribution is altered or targeted toward sites of inflammation in disease-specific models. Because vasodilation and increased vascular permeability are critical early steps in the inflammatory response, it is possible that MBV might distribute preferentially to inflamed tissues, hence contributing to their apparent tissue and systemic effect in vivo.

The findings of the present study show that the immunomodulatory properties of MBV may have therapeutic benefit in the treatment of influenza-mediated pulmonary inflammation with applicability to other viral-mediated inflammatory diseases such as SARS-CoV-2. The anti-inflammatory and immunomodulatory properties of ECM biomaterials and, specifically, the MBV component of ECM biomaterials have been well documented. The expanded clinical applications of MBV in the management of influenza-mediated inflammatory pathology represent a worthwhile pursuit of future investigation.

## MATERIALS AND METHODS

### Experimental design

The objective of the present study was to investigate the role of systemic MBV administration in the treatment of influenza-mediated systemic cytokine storm and local pulmonary inflammation. Briefly, mice were infected with either 100 plaque-forming units of influenza A PR/8/34 H1N1 (in 50 μl of sterile PBS) or vehicle (50 μl of sterile PBS) by oropharyngeal aspiration. Mice were then injected with either 100 μl of 1 × 10^12^ particles of MBV/ml or vehicle (100 μl of sterile PBS) by tail vein injection on days 2 and 6 after influenza infection. Following treatment, lungs and spleens were collected from animals for further analysis using multiplex immunoassays, flow cytometry, quantitative polymerase chain reaction (qPCR), and histopathology. Further details of these methods are outlined below. Animal studies were conducted with the approval of the University of Pittsburgh Institutional Animal care and Use Committee (protocol 20077581).

### Preparation of acellular urinary bladder ECM

Urinary bladder ECM (UB-ECM) was prepared from market-weight pigs as previously described (*19*). Porcine urinary bladders from market-weight animals (approximately 110 kilograms) were acquired from Animal Biotech Industries, and the tunica serosa, tunica muscularis externa, and most of the tunica submucosa and tunica muscularis mucosa of the bladders were mechanically removed. The luminal urothelial cells of the tunica mucosa were then dissociated from the basement membrane by washing with deionized water. The remaining tissue consisted of basement membrane, subjacent lamina propria of the tunica mucosa, and remnants of the tunica submucosa. This tissue was decellularized and digested by agitation in 0.1% peracetic acid with 4% ethanol for 2 hours at 300 rpm. The tissue was then extensively rinsed with 1× PBS (pH 7.4) and sterile deionized water. Last, the UB-ECM was lyophilized and milled into particulate using a Wiley Mill with a #40 mesh screen.

### Isolation of MBVs

MBVs were isolated from laboratory-produced porcine UB-ECM by enzymatic digestion and validated for size, morphology, and surface marker expression as performed and characterized in prior studies (*18*, *20*, *26*). Enzymatic digestion was performed using Liberase TL (highly purified collagenase I and collagenase II) in buffer [50 mM tris (pH 7.5), 5 mM CaCl_2_, and 150 mM NaCl] for 24 hours at room temperature on an orbital rocker. Digested UB-ECM was then subjected to centrifugation at 10,000*g* for 30 min at 4°C and filtered through a 0.22-μm filter. The clarified supernatant containing the liberated MBV was then centrifuged at 100,000*g* (Beckman Coulter Optima L-90K Ultracentrifuge) at 4°C for 70 min to pellet the MBV. MBV were then resuspended in sterile 1× PBS (pH 7.4), and particle concentration was determined using particle nanotracking analysis.

### Influenza inoculation, treatment, and disease monitoring

Influenza A PR/8/34 H1N1 was propagated as previously described (*59*). Six- to 8-week-old male C57BL/6 mice were purchased from Taconic Biosciences (Germantown, NY) and infected with either 100 plaque-forming units of influenza A PR/8/34 H1N1 (in 50 μl of sterile PBS) or vehicle (50 μl of sterile PBS) by oropharyngeal aspiration. Mice were injected with either 100 μl of 1 × 10^12^ particles of MBV/ml or vehicle (100 μl of sterile PBS) by tail vein injection on days 2 and 6 after influenza infection.

### Lung processing

Mouse lungs were lavaged with 1 ml of sterile PBS for inflammatory cell counts, flow cytometry, and lung leak analysis. Extent of lung leak was determined by protein analysis of BAL fluid using the Pierce Coomassie (Bradford) Protein Assay Kit (Thermo Fisher Scientific, Waltham, MA). The left lobe of each animal was inflated and fixed in cold 10% neutral-buffered formalin solution (pH 7.4) and embedded in paraffin wax for histology. Slides were processed and sectioned by StageBio (Mount Jackson, VA). The superior lobe of the right lung was placed in sterile PBS on ice for flow cytometry processing and analysis. The inferior lobe of the right lung was homogenized in 1 ml of PBS for cytokine analysis by Bio-Plex Multiplex immunoassay (Bio-Rad, Hercules, CA).

### RNA isolation and qPCR

The middle lobe of the right lung was snap-frozen and homogenized in lysis buffer for RNA extraction using an RNA isolation kit (Agilent Technologies, Santa Clara, CA). RNA analysis was performed by reverse transcription PCR (RT-PCR) using FAM probe 5′-/56-FAM/CTCAGTTAT/ZEN/TCTGCTGGTGCAC TTGCCA/3IABkFQ/-3′; forward primer, 5′-GGACTGCAGCGTAGACGCTT-3′; and reverse primer, 5′-CATCCTGTTGTATATGAGGCCCAT-3′ (Integrated DNA Technologies, Coralville, IA) to measure influenza M protein expression relative to the housekeeping gene HPRT (catalog no. 4331182, Thermo Fisher Scientific, Waltham, MA) using delta-delta *C*_t_ analysis (*60*).

### Flow cytometry

Isolated cells were stained in two panels, T/NK (natural killer) cell panel and myeloid/B cell panel, using the following antibodies to the indicated molecules (clone no.) [T/NK cell panel: CD45(30-F11), CD3(145-2C11), CD4(GK1.5), CD8(53-6.7), ST2(U29-93), CD69(H1.2F3), CD62L(MEL-14), CD44(IM7), CD279(PD-1)(RMP1-30), TCRγδ(GL3), NK1.1(PK136), Tbet(O4-46), GATA3(L50-823), FoxP3(FJK-16s), and RORγT(Q31-378); myeloid/B cell panel: CD45R(RA3-6B2), CD11b(M1/70), CD11c(HL3) Ly6C(AL-21), Ly6G(1A8), SiglecF(E50-2440), IA/IE (class II) (2G9), F4/80(BM8), CD86(GL1), CD103(M290), CD64(X54-5/7.1), CD301b(LOM-14), ST2(U29-93), CD45(30-F11), iNOS (CXNFT), and CD206(C068C2)]. Data were acquired using an Aurora (Cytek Biosciences) and analyzed using FlowJo (Tree Star, Ashland, OR). Intracellular staining was carried out using Foxp3/Transcription Factor staining buffer set (eBioscience; San Diego, CA). Live/dead exclusion was completed using E506 Viability Dye (Invitrogen).

### Histopathology and QuPath analysis

The left lobe of each animal was inflated and fixed in 4C 10% neutral-buffered formalin solution (pH 7.4) and embedded in paraffin for histology. Slides were sectioned by StageBio (Mount Jackson, VA). Sections were stained with either hematoxylin and eosin (H&E) or Masson’s trichrome stain. Whole-lung sections were imaged at 40× magnification on a Motic Slide scanner. QuPath, an open-source image analysis software, was used to evaluate the whole slide images (*61*). QuPath uses a machine learning algorithm to classify pixels and identify objects within an image on the basis of a training dataset. In the present study, the training data were obtained by manual identification and classification of normal tissue and various pathologic changes in the H&E and Masson’s trichome stained slides. Briefly, QuPath was trained to identify healthy lung parenchyma compared to inflammatory involvement in H&E images. In the trichrome images, QuPath was trained to identify healthy lung parenchyma, repair parenchyma (repair phenotype), airways, and vasculature within Masson’s trichrome–stained images. Samples were then batch-processed to identify these characteristics within all samples in an automated fashion without user input.

### Statistical analysis

Significant differences were determined at *P* < 0.05. All values are represented as means ± SEM. QuPath analysis of histopathology represents an *n* of 4 for Control + intravenous PBS group, an *n* of 7 for the H1N1 + intravenous PBS group, and an *n* of 7 for the H1N1 + intravenous MBV group. Luminex data represent an *n* of 4 for Control + intravenous PBS group, an *n* of 8 for the H1N1 + intravenous PBS group, and an *n* of 8 for the H1N1 + intravenous MBV group. Flow cytometry data represents an *n* of 4 animals for all groups. Differences among groups were determined using one-way analysis of variance (ANOVA) with Tukey’s post hoc correction with no adjustments made for multiplicity. All replicate values are plotted in addition to means ± SEM. All data were analyzed using Prism 9 (GraphPad).

## Notes

View/request a protocol for this paper from *Bio-protocol*.
