# Supplementary material for: Mitigation of influenza-mediated inflammation by immunomodulatory matrix-bound nanovesicles
Source: Sci Adv. 2023 May 19;9(20):eadf9016. doi: 10.1126/sciadv.adf9016 (PMC10198633; doi:10.1126/sciadv.adf9016)
Supplement: Supplementary file 1 — Figs. S1 to S4 Tables S1 to S3 [file sciadv.adf9016_sm.pdf]

Science Advances
AAAS

Supplementary Materials for

Mitigation of influenza-mediated inflammation by immunomodulatory matrix-bound nanovesicles

Raphael J. Crum *et al.*

Corresponding author: Stephen F. Badylak, badylaks@upmc.edu

Sci. Adv. **9**, eadf9016 (2023)
DOI: 10.1126/sciadv.adf9016

This PDF file includes:

Figs. S1 to S4
Tables S1 to S3

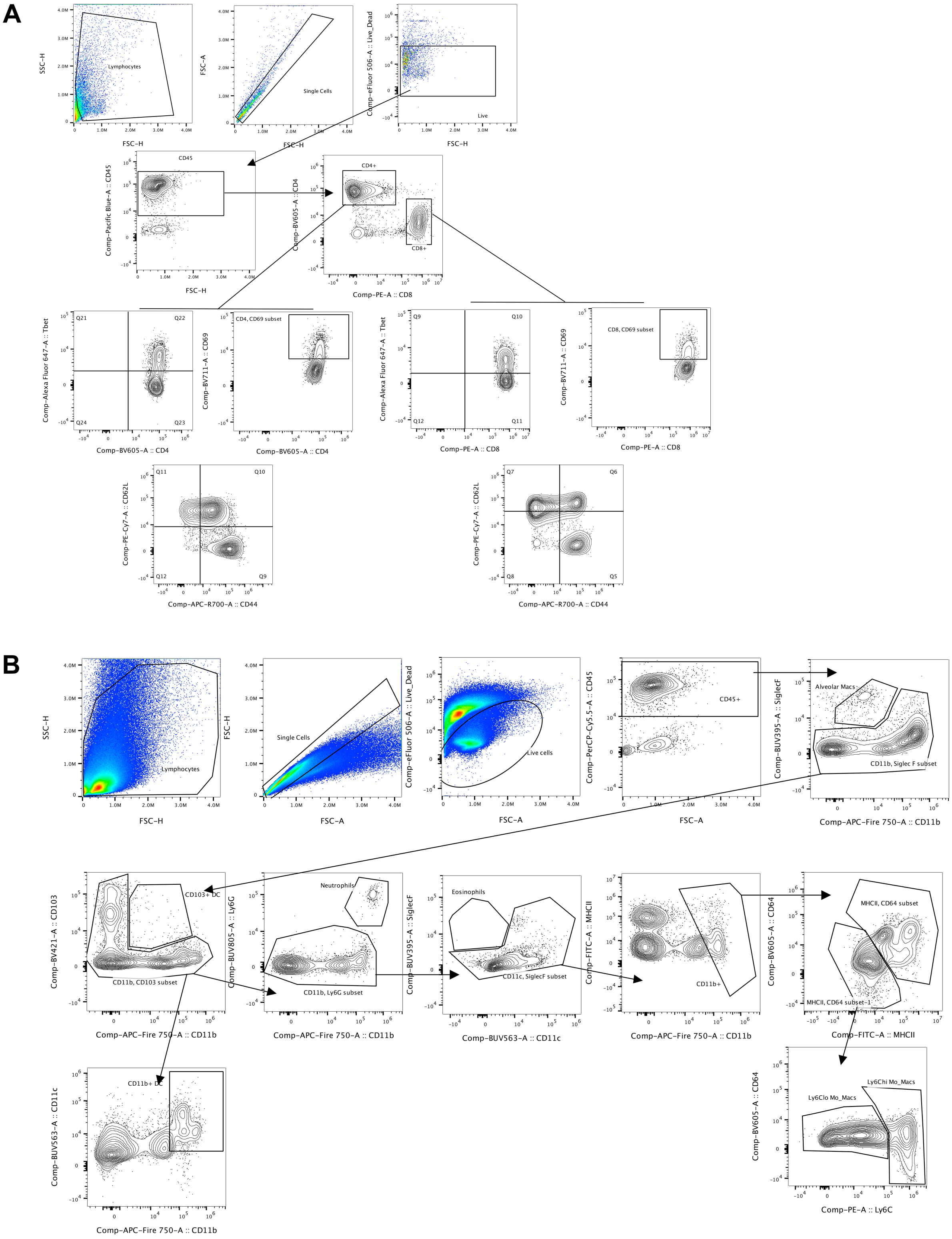
A
SSC-H
FSC-H
Lymphocytes
FSC-A
Single Cells
Comp-eFluor 506-A :: Live_Dead
Live
Comp-Pacific Blue-A :: CD45
CD45
Comp-BV605-A :: CD4
CD4+
CD8+
Comp-PE-A :: CD8
Comp-Alexa Fluor 647-A :: Tbet
Q21
Q22
Q23
Q24
Comp-BV711-A :: CD69
CD4, CD69 subset
Q9
Q10
Q11
Q12
CD8, CD69 subset
Comp-PE-Cy7-A :: CD62L
Comp-APC-R700-A :: CD44
Q5
Q6
Q7
Q8
B
Lymphocytes
Single Cells
Live cells
FSC-A
Comp-PerCP-Cy5.5-A :: CD45
CD45+
Comp-BUV395-A :: SiglecF
Alveolar Macs
CD11b, Siglec F subset
Comp-APC-Fire 750-A :: CD11b
Comp-BV421-A :: CD103
CD103+ DC
CD11b, CD103 subset
Comp-BUV805-A :: Ly6G
Neutrophils
CD11b, Ly6G subset
Eosinophils
CD11c, SiglecF subset
Comp-BUV563-A :: CD11c
Comp-FITC-A :: MHCII
CD11b+
Comp-BV605-A :: CD64
MHCII, CD64 subset
MHCII, CD64 subset-1
CD11b+ DC
Ly6Clo Mo_Macs
Ly6Chi Mo_Macs
Comp-PE-A :: Ly6C

Figure S1: Flow cytometry gating strategy. A-B). $CD45^+$ cells from the spleen and lung were assessed by flow cytometry on d7 and d21. **A.**) Representative flow plot gating to identify lung $CD45^+CD4^+$ and $CD45^+CD8^+$ T cells. **B.)** Representative flow plot gating to identify lung $CD45^+CDD11b^{Hi}Ly6G^+$ neutrophils, CD11bInt $SiglecF^+$ alveolar macrophages, CD11lb-Hi $CD64^-$ $MHCII^-$ Ly6C-Hi monocytes and $CD11b^+$ CD11c+ dendritic cells.

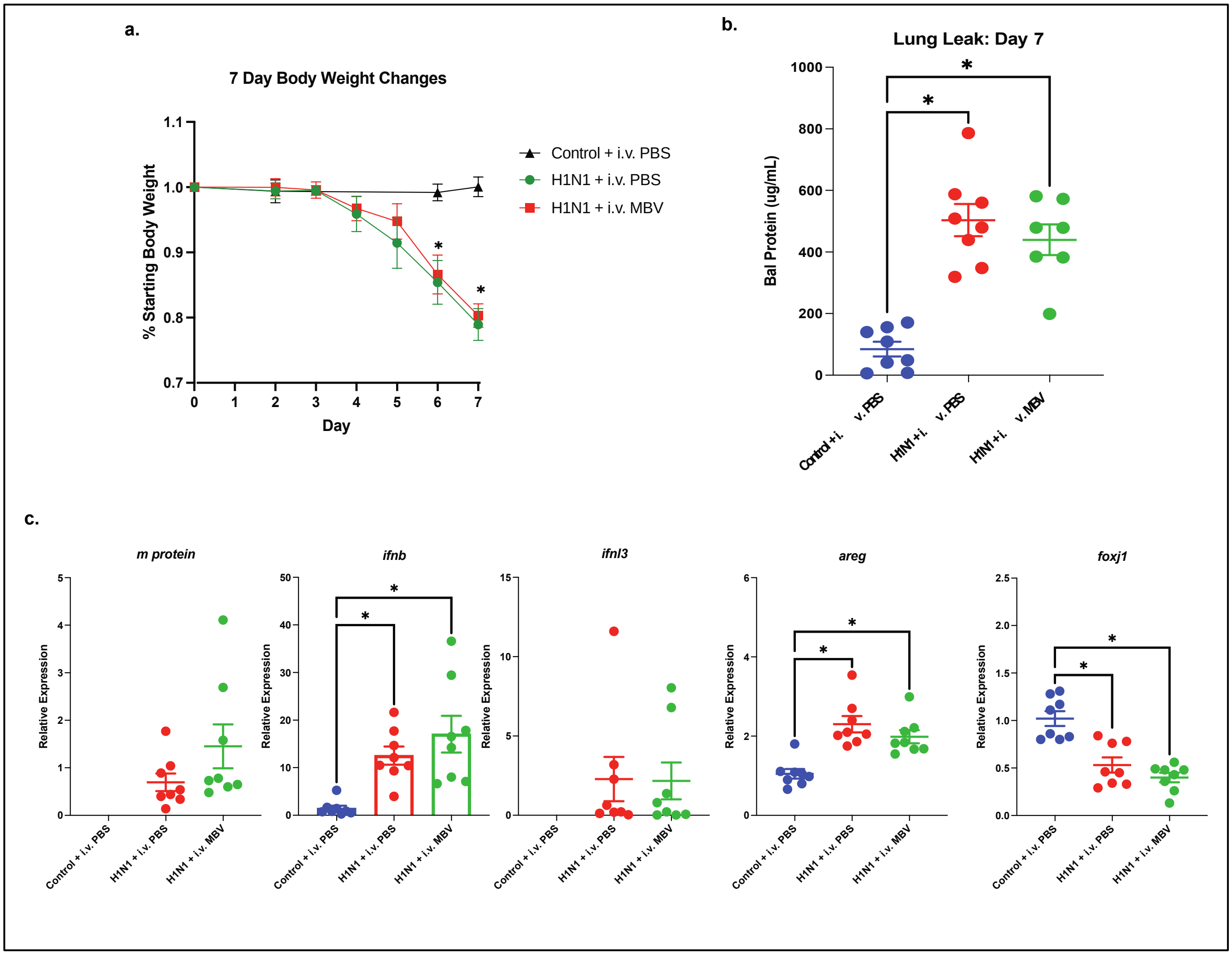

Figure S2: Body weight, lung leak, and gene expression from 7-day survival group. a.) Body weight changes in each group throughout the 21-day study. Weight change was quantified as percent of starting body weight. n = 8. b.) Lung leak was measured by quantifying the total protein concentration in bronchiolar lavage fluid (BAL; µg/mL) c.) Expression of genes associated with IFN β and λ signaling and epithelial-related genes were assessed using qPCR. n=8. All values are represented as mean ± SEM and significant differences * = p<0.05.

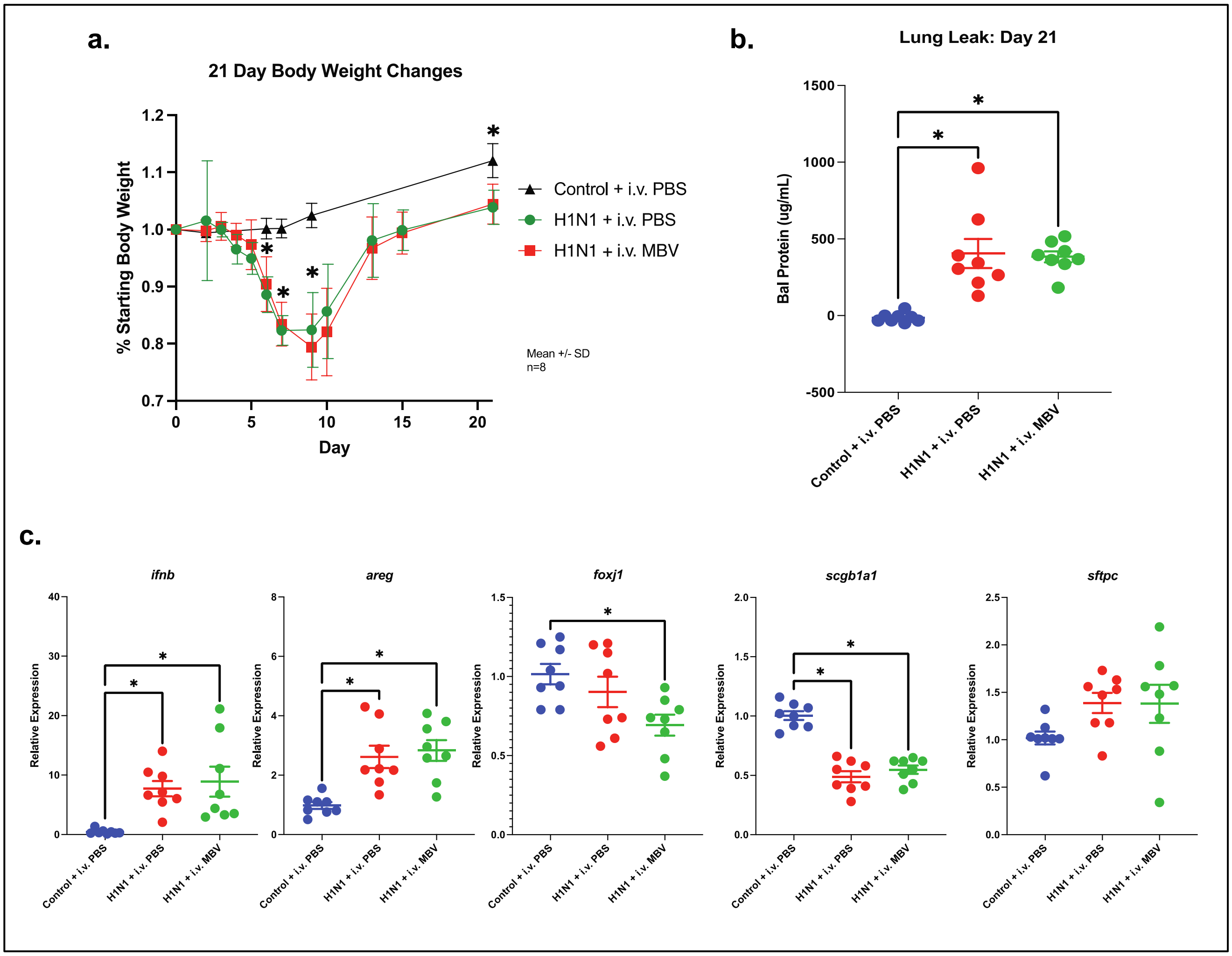

Figure S3: Body weight, lung leak, and gene expression from 21-day survival group. a.) Body weight changes in each group throughout the 21-day study. Weight change was quantified as percent of starting body weight. n = 8. **b.)** Lung leak was measured by quantifying the total protein concentration in bronchiolar lavage fluid (BAL; µg/mL) **c.)** Expression of genes associated with IFN β and λ signaling and epithelial-related genes were assessed using qPCR. n=8. All values are represented as mean ± SEM and significant differences * = p<0.05.

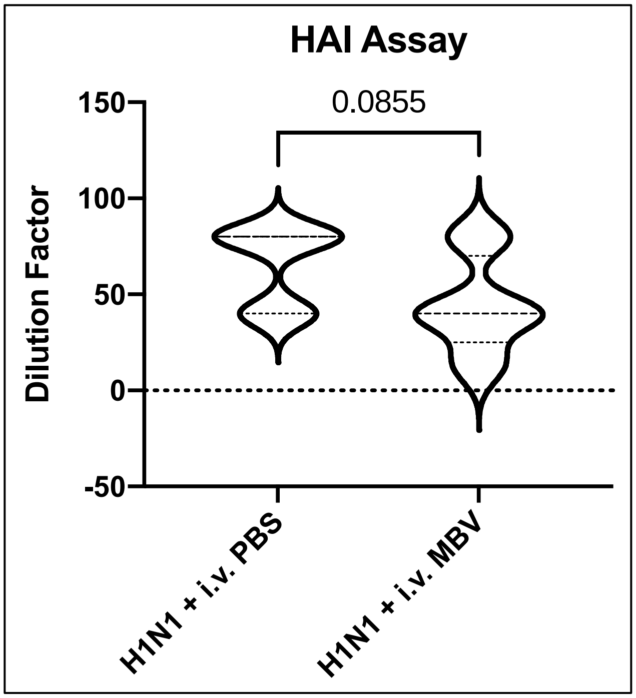

Figure S4: Influenza Hemagglutination-inhibition (HAI) antibody titers. HAI antibody titers were assessed in the H1N1 + i.v. PBS and H1N1 + i.v. MBV groups. n=8.

Table S1: Concentrations of cytokines and chemokines in lung homogenates at day 7.

Target Protein	Control + PBS	H1N1 + i.v. PBS	H1N1 + i.v. MBV	Significant differences (*p*<.05)
Eotaxin	50.73 ± 15.42	145.4 ± 46.78	79.54 ± 9.0	n.s.
GCSF	0.00 ± 0.00	110.0 ± 30.03	32.95 ± 11.48	
IL-12p70	181.2 ± 24.69	245.6 ± 21.68	205.9 ± 21.09	n.s.
IL-13	214.8 ± 13.53	282.5 ± 18.11	255.7 ± 13.80	Yes, Control + PBS vs H1N1 + PBS
IL-3	8.63 ± 1.08	16.91 ± 1.29	13.77 ± 1.19	Yes, Control + PBS vs H1N1 + PBS; Control + PBS vs H1N1 + MBV
IL-4	3.83 ± 1.03	5.99 ± 0.63	4.08 ± 0.72	n.s.
MCP-1	86.48 ± 19.93	4036.00 ± 879.90	2250.00 ± 369.20	Yes, Control + PBS vs H1N1 + PBS
MIP1a	8.30 ± 2.20	455.8 ± 96.01	242.7 ± 46.90	Yes, Control + PBS vs H1N1 + PBS
GMCSF	42.78 ± 12.57	78.37 ±6.64	54.06 ±10.60	n.s.

IL-5	13.18 ± 1.20	35.58 ± 3.88	26.37 ± 3.11	Yes, Control + PBS vs H1N1 + PBS
IL-6	24.84 ± 4.72	72.72 ± 9.69	42.89 ± 3.73	Yes, Control + PBS vs H1N1 + PBS; H1N1 + PBS vs H1N1 + MBV
MIP1b	212.20 ± 22.60	1823.00 ± 354.90	1087.00 ± 130.60	Yes, Control + PBS vs H1N1 + PBS
IFNγ	42.99 ± 7.34	129.00 ± 19.04	67.18 ± 11.41	Yes, Control + PBS vs H1N1 + PBS; H1N1 + PBS vs H1N1 + MBV
IL-1α	18.93 ± 1.99	25.75 ± 2.70	20.09 ± 2.82	n.s.
IL-9	57.02 ± 6.51	84.28 ± 7.09	62.33 ± 9.98	n.s.
RANTES	607.70 ± 208.40	3012.00 ± 591.90	2110 ± 240.90	Yes, Control + PBS vs H1N1 + PBS
IL-12p40	193.90 ± 22.00	520.00 ± 65.41	409.30 ± 40.01	Yes, Control + PBS vs H1N1 +

				PBS; Control + PBS vs H1N1 + MBV
IL-2	20.24 ± 1.81	22.60 ± 1.23	19.58 ± 2.09	n.s.
KC	25.92 ± 3.18	176.40 ± 16.75	86.20 ± 13.71	Yes, Control + PBS vs H1N1 + PBS; Control + PBS vs H1N1 + MBV; H1N1 + PBS vs H1N1 + MBV
IL-10	38.02 ± 5.76	89.95 ± 7.49	64.51 ± 6.14	Yes, Control + PBS vs H1N1 + PBS; H1N1 + PBS vs H1N1 + MBV
IL-1β	4.76 ± 0.46	13.85 ± 1.95	8.83 ± 0.98	Yes, Control + PBS vs H1N1 + PBS; H1N1 + PBS vs H1N1 + MBV
IL-17	21.87 ± 2.28	20.38 ± 0.77	16.42 ± 2.22	n.s.

TNFα	189.60 ± 27.58	287.10 ± 18.41	217.70 ± 22.43	Yes, Control + PBS vs H1N1 + PBS
Values are represented as Mean ± Standard Error of Mean.				

Table S2: Concentrations of cytokines and chemokines in lung homogenates at day 21.

Target Protein	Control + PBS	H1N1 + i.v. PBS	H1N1 + i.v. MBV	Significant differences (*p*<.05)
Eotaxin	27.67 ± 6.23	44.46 ± 4.68	29.76 ± 4.48	n.s.
GCSF	0.00 ± 0.00	5.31 ± 2.93	0.00 ± 0.00	n.s.
IL-12p70	181.2 ± 24.69	245.6 ± 21.68	205.9 ± 21.09	n.s.
IL-13	199.10 ± 32.58	302.70 ± 30.32	241.60 ± 15.09	n.s.
IL-3	6.57 ± 1.68	16.25 ± 2.39	7.89 ± 1.47	Yes, Control + PBS vs H1N1 + PBS; H1N1 + PBS vs H1N1 + MBV
IL-4	1.21 ± 0.71	3.38 ± 0.61	2.72 ± 0.69	n.s.
MCP-1	39.51 ± 22.83	266.5 ± 58.77	71.86 ± 15.18	Yes, Control + PBS vs H1N1 + PBS; H1N1 + PBS vs H1N1 + MBV
MIP1a	4.60 ± 0.68	11.95 ± 1.34	9.30 ± 1.19	Yes, Control + PBS vs H1N1 + PBS

GMCSF	22.75 ± 13.32	67.73 ± 9.47	50.03 ± 4.79	Yes, Control + PBS vs H1N1 + PBS
IL-5	9.36 ± 2.98	28.00 ± 4.78	15.19 ± 2.28	Yes, Control + PBS vs H1N1 + PBS; H1N1 + PBS vs H1N1 + MBV
IL-6	10.62 ± 4.19	18.49 ± 2.96	19.95 ± 4.61	n.s.
MIP1b	106.4 ± 42.93	227.90 ± 30.48	160.50 ± 36.12	n.s.
IFNγ	25.58 ± 9.89	41.78 ± 4.12	35.24 ± 2.47	n.s.
IL-1α	16.73 ± 4.47	16.49 ± 1.17	13.84 ± 1.81	n.s.
IL-9	31.94 ± 9.06	65.43 ± 6.57	55.35 ± 3.60	Yes, Control + PBS vs H1N1 + PBS
RANTES	266.5 ± 46.86	717.5 ± 80.53	452.1 ± 74.03	Yes, Control + PBS vs H1N1 + PBS; H1N1 + PBS vs H1N1 + MBV
IL-12p40	122.20 ± 29.70	484.80 ± 58.45	345.90 ± 24.16	Yes, Control + PBS vs H1N1 + PBS; Control +

				PBS vs H1N1 + MBV
IL-2	16.30 ± 2.47	18.84 ± 0.92	15.93 ± 1.71	n.s.
KC	18.90 ± 4.06	176.30 ± 57.68	31.71 ± 4.42	Yes, H1N1 + PBS vs H1N1 + MBV
IL-10	20.27 ± 10.96	56.81 ± 8.06	39.55 ± 3.46	Yes, Control + PBS vs H1N1 + PBS
IL-1β	3.49 ± 1.12	10.23 ± 1.53	4.15 ± 0.65	Yes, Control + PBS vs H1N1 + PBS; H1N1 + PBS vs H1N1 + MBV
IL-17	15.57 ± 4.88	20.02 ± 1.72	17.53 ± 2.08	n.s.
TNFα	119.60 ± 23.10	211.70 ± 13.70	186.70 ± 10.09	Yes, Control + PBS vs H1N1 + PBS; Control + PBS vs H1N1 + MBV
Values are represented as Mean ± Standard Error of Mean.				

Table S3: Antibodies used for flow cytometry

Clone	Marker	Fluorophore	Dilution
	Zombie Live/Dead	e506	
30-F11	CD45	PB	1:200
145-2C11	CD3	BV650	1:200
GK1.5	CD4	BV605	1:300
53-6.7	CD8	PE	1:100
U29-93	ST2	BUV737	1:200
H1.2F3	CD69	BV711	1:300
MEL-14	CD62L	PeCy7	1:400
IM7	CD44	APC-R700	1:300
RMP1-30	PD-1	BUV395	1:300*
GL3	gdTCR	APC-Fire 750	1:300*
PK136	NK.1.1	FITC	1:300*
O4-46	Tbet	AF647	1:50
L50-823	Gata3	PE-CF594	1:50
FJK-16s	Foxp3	PercpCy5.5	1:50
Q31-378	Rorgt	BV421	1:50
RA3-6B2	B220	PeCy5	1:200
M1/70	CD11b	APC Fire 750	1:100
HL3	CD11c	BUV563	1:100
AL-21	Ly6C	PE	1:200
1A8	Ly6G	BUV805	1:200

E50-2440	SiglecF	BUV395	1:100
2G9	MHCII	FITC	1:200
BM8	F4-80	PacBlue	1:100
GL1	CD86	BV650	1:200
M290	CD103	BV421	1:200
X54-5/7.1	CD64	BV605	1:100
LOM-14	CD301b Mgl2	PE-CF594	1:200
U29-93	ST2	BUV737	1:100
30-F11	CD45	PercpCy5.5	1:200
CXNFT	iNOS	PeCy7	1:300
C068C2	CD206	AF647	1:300
